# A clinical and biochemical comparative study of diabetic ketoacidosis (DKA) in newly diagnosed vs known cases of Type 1 diabetic children

**DOI:** 10.1900/RDS.2023.19.28

**Published:** 2023-03-31

**Authors:** Waleed H. Albuali, Abdullah A. Yousef, Mohammad H. Al-Qahtani, Faisal O. AlQurashi, Hamad W. Albuali, Haneen A. Yousef, Ala’a A. Aldajani, Mohammed A. Al Ghamdi, Bassam N. AlBassam

**Affiliations:** 1 Department of Pediatrics, College of Medicine, King Fahd Hospital of the University, Imam Abdulrahman Bin Faisal University, Dammam, Saudi Arabia,; 2 Department of Family and Community Medicine, College of Medicine, Imam Abdulrahman Bin Faisal University, Dammam, Saudi Arabia.

**Keywords:** diabetic ketoacidosis, newly diagnosed, biochemical profile, Type 1 diabetes mellitus, pediatric intensive care unit (PICU)

## Abstract

**Objectives:**

We aimed to study the characterizing clinical and biochemical profiles of Diabetic Ketoacidosis (DKA) in children with newly diagnosed Type 1 Diabetes Mellitus (Type 1DM) compared to children with established diagnosis of Type 1DM presenting with DKA admitted to the pediatric intensive care unit of a large university hospital in the eastern region of Saudi Arabia.

**Methods:**

We retrospectively reviewed the medical records of 211 patients who were admitted to the pediatric intensive care unit with diabetic ketoacidosis between 2010 and 2019. The diagnosis of diabetic ketoacidosis was based on symptoms of polydipsia, polyurea, weight loss, vomiting, dehydration, abdominal pain, breathing problems, lethargy or coma, biochemical hyperglycemia (blood glucose level of >200 mg/dL), venous pH of <7.3, serum bicarbonate level of ≤15 mEq/L, and ketonemia (blood β-hydroxybutyrate concentration of ≥3 mM) or moderate or severe ketonuria (diagnosed as newly acquired type 1 diabetes).

**Results:**

The rate of newly diagnosed Type 1 DM with DKA was 41.7%, out of them who got severe and moderate diabetic ketoacidosis were 61.6% and 38.4%, respectively. We observed significantly increased heart and respirator y rates in patients newly diagnosed with diabetic ketoacidosis and in those with severe diabetic ketoacidosis (p<0.001) compared to known cases with Type 1DM presenting with DKA. We also identified significantly increased biochemical indices including HbA1c, random blood sugar, serum osmolality, blood urea nitrogen, creatinine, chloride, lactate, and anion gap in relation to severe diabetic ketoacidosis and newly diagnosed type 1 diabetes (p ≤ 0.05).

**Conclusions:**

We found that the clinical and biochemical profiles of patients with newly diagnosed Type 1 DM children were significantly affected compared to children who were known Type 1DM presenting with DKA.

## 1. Introduction

Type 1 diabetes mellitus (Type 1DM) is the most common type of diabetes in children with about 90% prevalence [1] and is caused by a decrease or loss of production of insulin as a result of autoimmune destruction of the β pancreatic cells in the islets of Langerhans. Therefore, insulin supplementation through multiple daily injections or continuous subcutaneous insulin infusion pumps is needed for survival in patients with type 1 DM [2,3]. Diabetic ketoacidosis (DKA) is an acute complication of type 1 DM that may lead to severe morbidity and a life-threatening state of the disease, which might happen the first clinical presentation of newly diagnosed Type 1 diabetic children or might happen for the known cases of Type diabetic children through there life as a consequence of different etiologies [3,4].The incidence of DKA in newly diagnosed Type diabetic children varies dramatically among countries from 13 to 80 % inversely correlated to the prevalence of Type 1 DM in that country, and it is less common in adolescents compared to young children [5]. In Saudi Arabia the incidence of DKA in newly diabetic children [6,7].

The major causes of intermittent DKA in the known diabetics includes missing insulin doses or underdosing, cellular insulin pump dysfunctions, infections, as well as prolonged stress [8,9].

The international incidence of DKA in known cases of Type 1 DM is approximately 5–7% in pediatric patients [10]. A study reported that the most common precipitating factors of DKA were infection and poor compliance to treatment (54.4% and 28.0%, respectively) [11].

Significantly decreased levels of biochemical markers, including blood glucose, pH, bicarbonate anion (HCO_3_^-^), glycated hemoglobin (HbA1c), vitamin D, thyroid-stimulating hormone, free thyroxine, Ca^2+^, creatinine, Na^+^, K^+^, and lactate levels were reported in the presence of DKA [12,13]. However, both these studies did not compare biochemical concentrations in relation to DKA in pediatric patients newly diagnosed with type 1 DM vs. DKA exacerbations in pediatric patients with known type 1 diabetes, before and after treatment in regard to the severity of DKA in the two groups.

In a recent study, a significantly higher incidence of severe DKA was reported in children below as compared to above 5 years of age (21.8% vs. 3.75%; p=0.021), which accentuates the need to increase awareness and prevent DKA among parents with DM [14]. More encouraging results are expected to be obtained by involving regular and continuous educational programs for medical practitioners as well as parents or caregivers, in addition to strategies for glycemic control, periodic monitoring of laboratory profiles, and engagement of children in physical activity [15].

This retrospective study aimed at comparing the clinical and biochemical profile of the first DKA exacerbation between children with newly diagnosed type 1 diabetes and children with known type 1 diabetes. This comparison was made in relation to the severity of each patient’s condition and using biomarkers of ten years’ worth of data from the pediatric intensive care unit (PICU) of a university hospital. With this study, we have aimed at improving the clinicians’ awareness of the need for periodic monitoring of clinical and biochemical profiles for the early assessment of DKA. Thereby, health professionals may plan treatment more efficiently in addition to continued diabetic education and screening campaigns. Such endeavors are expected to assist known pediatric diabetes mellitus patients to minimize the burden of DKA exacerbations.

## 2. Material and Methods

### 2.1 Aim

This retrospective longitudinal study aimed to compare the clinical and biochemical profiles between the 1st DKA exacerbation in newly diagnosed children with type 1 diabetes and the DKA exacerbations of children with known type 1 diabetes admitted to the PICU.

### 2.2 Participants

Pediatric patients aged <14 years admitted to the PICU of King Fahd University Hospital (KFUH) with a diagnosis of DKA during a period of ten years from 2010 to 2019 were included in this study. The PICU of the KFUH is a 10-bed multidisciplinary unit that is well equipped to accommodate infants and children. The unit provides specialized care to patients with complex, surgical, oncological, orthopedic, and traumatic care requirements. It serves a community of ~4.9 million people with approximately 250 admissions annually. Patients with incomplete or missing data were excluded from this study.

### 2.3 Data

Complete medical records of known pediatric patients with Type 1 DM for more than 1 year duration with DKA having one or more episodes in the previous year.

Also, we included pediatric patients newly diagnosed with Type 1 DM who presented with polydipsia, polyurea, abdominal pain, vomiting, dehydration, weight loss, breathing disturbances due to metabolic acidosis, central nervous system conditions such as lethargy or coma, and elevated blood glucose level of >200 mg/dL and had been admitted to the PICU from 2010 to 2019 were retrieved. Patients with known type 1 diabetes presenting with DKA symptoms were also included. DKA was defined according to the International Society for Pediatric and Adolescent Diabetes guidelines 2018 [16] by patients bearing blood glucose of >11 mM (>200 mg/dL), ketonuria and/or ketonemia, and a venous pH value of <7.3. The severity of DKA was classified as mild (7.2≤ pH <7.3 or bicarbonate anion concentration of 10–15 mM), moderate (7.1≤ pH <7.2 and bicarbonate anion concentration of 5–10 mM), or severe (pH <7.1 and bicarbonate anion concentration of <5 mM). Cases without DKA were defined by bicarbonate anion concentrations of ≥15 mM or pH ≥7.30. The patients’ clinical features in terms of hemodynamic responses including systolic blood pressure (SBP), diastolic blood pressure (DBP), heart rate (HR), and respiratory rate were recorded on a digital monitoring device in the PICU. The biochemical profile included HbA1c, random blood sugar (RBS), serum osmolality, blood urea nitrogen (BUN), creatinine, Na^+^, K^+^, Cl^-^, phosphorus, pH, PCO_2_, HCO_3_, lactate, and anion gap retrieved from hospital laboratory records at the time of admission for all patients.

This study was approved by the Institutional Review Board (IRB) of Imam Abdulrahman Bin Faisal University (IRB-2021-01-235). The IRB waived the need for obtaining informed consent because of the retrospective nature of the study. The datasets used and/or analyzed in the current study are available from the corresponding author upon request.

### 2.4 Analysis

The data analysis was performed using SPSS version 22 (IBM Corp., Chicago, IL, USA). Categorical variables including sex and known or newly diagnosed DKA are presented as frequencies using percentages. Continuous data variables such as biomarkers and length of PICU stay are presented as mean±standard deviation. They were found to follow normal distributions using one-sample Kolmogorov– Smirnov tests. Unpaired t-tests were used to compare these variables between known vs. newly diagnosed DKA and moderate versus severe DKA. Multivariate regression analysis was performed by considering the type of DKA (known/new) and severity of DKA as dependent variables, whereas demographic, clinical, and biochemical profile covariates were considered as independent variables. Statistical significance was set to p≤0.05.

## 3. Results

Of 211 children admitted with a diagnosis of DKA, 120 (56.9%) were male and 91 (43.1%) were female. The average age of the children was 7.74±4.24 years (ranging 0–14 years). There were 123 (58.3%) known and 88 (41.7%) new cases of DKA which revealed a 41.7% rate of newly diagnosed DKA in our study. The severity of DKA was moderate in 81 (38.4 %) patients and severe in 130 (61.6%). Of 88 patients with newly diagnosed DKA, 57 had severe DKA, which revealed a severe DKA rate of 64.8%.

Significantly increased RR and SBP were found in patients with newly diagnosed DKA (p=0.006 and p=0.019, respectively). HbA1c and RBS of all children were significantly higher than normal revealing poor glycemic control in both the known and newly diagnosed groups. The average HbA1c, RBS, BUN, creatinine, K^+^, Cl^-^, phosphorus, and lactate levels were higher in patients newly diagnosed with DKA at p≤0.05, as presented in Table 1. Significantly increased HR and RR were observed in patients with severe DKA (p<0.001). Significantly elevated HbA1c, RBS, serum osmolality, BUN, creatinine, Cl^-^, lactate, and anion gap were observed in severe DKA as compared to moderate DKA (p≤0.05; Table 2).

**Table 1.** Mean differences of clinical and biochemical indices between known and newly diagnosed DKA

Variables	Known (n=123)	New case (n=88)	p-value
**HR/min**	137.98±14.14	135.38±22.50	0.304
**RR/min**	32.07±5.74	34.14±4.72*	0.006
**SBP mm Hg**	98.50±8.77	101.76±11.26*	0.019
**DBP mm Hg**	52.85±9.26	53.01±6.65	0.892
**HbA1c %**	12.11±1.30*	9.77±1.62	<0.001
**Random blood sugar mg/dl**	486.59±50.16	429.13±69.88*	<0.001
**Serum Osmolality mOsm/Kg**	289.81±5.07	289.98±6.39	0.836
**Blood urea nitrogen mg/dL**	16.16±4.88	20.77±6.54*	<0.001
**Creatinine mg/dL**	0.98±0.31	1.44±0.82*	<0.001
**Sodium mmol/L**	132.85±1.98	132.91±2.00	0.819
**Potassium mmol/L**	3.46±0.17	3.60±0.39*	0.001
**Chloride mmol/L**	93.08±4.21	90.48±4.11*	<0.001
**Phosphorus mg/dL**	3.27±0.34	3.39±0.38*	0.018
**pH**	7.06±0.08	7.06±0.12	0.922
**PCO_2_ mmHg**	21.98±2.97*	19.18±4.65	<0.001
**HCO3 mmol/L**	7.21±2.95	6.95±3.33	0.545
**Lactate mmol/L**	2.30±0.79	3.63±1.67*	<0.001
**Anion gap. mmol/L**	19.12±3.03	19.06±3.56	0.886

* , significant at p≤0.05

**Table 2.** Mean differences of clinical and biochemical indices between moderate and severe DKA in children with type 1 DM

Variables	Moderate (n=81)	Severe (n=130)	p-value
**HR/min**	128.57±11.79	142.08±19.40*	<0.001
**RR/min**	30.62±4.73	34.38±5.34*	<0.001
**SBP mm Hg**	105.86±7.77*	96.12±9.39	<0.001
**DBP mm Hg**	57.75±6.03*	49.91±8.04	<0.001
**HbA1c%**	10.80±1.93	11.34±1.77*	0.038
**Random blood sugar mg/dL**	417.94±43.27	490.46±61.56*	<0.001
**Serum osmolality**	289.68±3.85	290.01±6.53*	0.682
**Blood urea nitrogen mg/dL**	15.95±4.21	19.41±6.64*	<0.001
**Creatinine mg/dL**	0.76±0.16	1.43±0.66*	<0.001
**Sodium mmol/L**	134.11±0.92*	132.10±2.08	<0.001
**Potassium mmol/L**	3.61±0.32*	3.46±0.26	<0.001
**Chloride mmol/L**	91.17±4.87	92.51±3.93*	0.030
**Phosphorus mg/dL**	3.43±0.32*	3.25±0.36	<0.001
**pH**	7.15±0.05*	7.01±0.09	<0.001
**PCO_2_ mmHg**	22.31±2.07*	19.88±4.60	<0.001
**HCO_3_ mmol/L**	10.66±1.96*	4.89±0.67	<0.001
**Lactate mmol/L**	2.19±1.24	3.27±1.33*	<0.001
**Anion gap. mmol/L**	17.33±2.82	20.19±3.02*	<0.001

* , significant at p≤0.05

The mean length of PICU stay was also significantly different between the newly diagnosed and the known DKA (4.72±2.82 vs. 3.24±1.69, respectively; p<0.001), as well as between severe and moderate DKA (4.53±2.72 vs. 2.77±0.73, respectively; p<0.001).

Multivariate regression analysis revealed a significant difference between the known or newly diagnosed DKA with regards to demographic, clinical, and biochemical parameter covariates, except for Cl^-^ (p=0.060) and HCO_2_ (p=0.485). Significant differences between moderate and severe DKA were also observed with regards to demographic, clinical, and biochemical parameter covariates, albeit not in terms of sex (p=0.084) and lactate level (p=0.215; Table 3).

**Table 3.** Multivariate regression analysis

Variables	Known/New diagnosis		Moderate/Severe DKA	
	β±S.E.	p-value	β±S.E.	p-value
**Age (years)**	-0.001±0.009**	0.002	-0.050±0.009**	0.001
**Sex**	-0.194±0.068**	0.004	0.117±0.067	0.084
**Weight (kg)**	0.018±.002**	0.008	-0.021±0.002**	0.001
**BMI (kg/m^2^)**	-0.045±.008*	0.041	0.079±0.007**	0.001
**HR (min)**	0.003±0.041**	0.005	-0.010±0.002**	0.001
**RR /min**	0.043±0.005**	0.003	-0.035±0.005**	0.001
**SBP mm Hg**	0.015±0.003**	0.003	-0.015±0.003**	0.001
**DBP mm Hg**	0.002±0.003*	0.013	-0.013±0.003**	0.001
**HbA1c%**	-0.193±0.013**	0.000	0.111±0.012**	0.001
**Random blood sugar mg/dl**	-0.002±0.000**	0.004	0.002±0.000**	0.001
**Serum Osmolality**	0.028±0.004**	0.003	-0.018±0.005**	0.001
**Blood urea nitrogen**	0.012±0.003*	0.030	0.014±0.003**	0.001
**Creatinine mg/dL**	0.156±0.030*	0.015	0.095±0.033**	0.004
**Sodium mmol/L**	0.114±0.015	0.063	-0.111±0.015**	0.001
**Potassium mmol/L**	0.664±0.063**	0.004	-0.320±0.066**	0.001
**Chloride mmol/L**	0.005±0.004	0.060	-0.020±0.005**	0.001
**Phosphorus mg/dL**	0.736±0.060**	0.002	-0.601±0.050**	0.001
**pH**	1.087±0.427*	0.012	-0.738±0.216**	0.001
**PCO_2_ mmHg**	-0.063±0.009**	0.000	0.015±0.005**	0.001
**HCO_3_ mmol/L**	0.009±0.013	0.485	-0.139±0.007**	0.000
**Lactate mmol/L**	0.163±0.022**	0.000	0.014±0.011	0.215
**Anion gap mmol/L**	-0.039±0.012**	0.001	-0.015±0.006*	0.015

* , significant at p≤0.05; **, significant at p≤0.01

## 4. Discussion

The rate of newly diagnosed DKA in our study was 41.7%, which was significantly higher than 28.8%, as reported in a recent study conducted in New Zealand, and was rather comparable to their last 5 years trend rate (27%) of DKA [15]. However, a similar rate of DKA (35.5%) was reported in an Indian youth diabetic registry from to 2006–2012 published in 2021 [17]. The recent outbreak of the Coronavirus disease 2019 pandemic seriously affected the rate of DKA (68.2% in 2020 vs. 45.6% in 2019), as reported in Canada [18]. The frequency of severe DKA in our study of 64.8% was significantly higher than that of the forementioned study where 27.1% and 13.2% incidence rates of severe DKA were observed in 2020 and 2019, respectively [18]. This finding is comparable to the study of Lovane et al. [14].

Dhatariya et al. reported a somewhat comparable incidence of DKA (33.7%), which is inconsistent with that of our study, yet they observed markedly different biochemical findings, that is, hypokalemia (55%) and hypoglycemia (27.6%) which are consistent with our study [19]. Fredheim et al. reported that moderate to severe DKA was associated with elevated mean HbA1c levels in severe/moderate versus mild/no DKA, respectively, i.e., 12.37±2.20 versus 11.21±2.33, [0.24; 95% confidence interval (CI) 0.11; 0.36; p=0.0003] and higher insulin dose-adjusted HbA1c (0.51; 95% CI 0.31; 0.70; p<0.0001) [20]. Our study also exhibited similar results, with an HbA1c mean of 11.34±1.77 in severe DKA being significantly higher than 10.80±1.93 in moderate DKA (p=0.038). Odeh et al. reported that HbA1c level correlated with the patients’ age and was significantly higher in patients of 11 years of age or older (12.0±1.9 vs. 11.2±20 and 10.3±1.8, respectively; p=0.001), whereas it did not differ by sex [21]. This indicates that poor glycemic control and higher HbA1c levels are more likely to result into severe DKA.

Lee et al. reported significantly decreased biochemical levels at the time of death vs. at the time of admission. SBP, DBP, creatinine level, and serum lactate level were found to be predictors of mortality [22]. In our study, significantly different HR and RR were observed in patients with severe DKA (p<0.001). Significantly elevated HbA1c, RBS, serum osmolality, BUN, creatinine, Cl^-^, lactate levels, and anion gap were observed in severe DKA compared to moderate DKA (p ≤ 0.05).

Notable changes during the initial 6–12 h of management regarding blood glucose, pH, anion gap, serum osmolality, serum K^+^, and serum phosphate have been reported [13]. We reported biochemical imbalance between known and newly diagnosed DKA, as well as between severe and moderate DKA. However, we could not report changes in response to management, as reported by Naeem et al. [13], which remains a limitation of our study. Nevertheless, a deductive approach would provide grounds to assume that biochemical levels were significantly imbalanced in severe DKA, and treatment response shifted the number of severe DKA based on the biochemical profile.

Notably, we observed elevated hemodynamics and intravenous fluid intake (p<0.05). The strict insulin dose compliance, general lifestyle, and physical activity of children with type 1 DM may reduce DKA exacerbations [23,24]. However, family awareness of potentially modifiable risk factors for new-onset DKA including prolonged delay of laboratory testing and suspicion of type 1 DM may lead to delayed diagnosis [25].

The limitations of our study; being a retrospective cohort study in the intensive care unit, in addition to its non-inclusive of all the possible aggravating factors of DKA in children from all the medical and non-medical aspects.

We conclude that the biochemical profile and clinical features of pediatric patients with severe DKA including heart and respiratory rates differed significantly between children with known and newly diagnosed type 1 diabetes.

Based on our results, we recommend more extensive health education and awareness for the public to detect early clinical signs and symptoms of type 1 diabetes in children to reduce the risk of having DKA as a presenting problem. In patients known to have type 1 diabetes, we recommend good glycemic control through strict appropriate insulin doses, periodic biochemical tests, balanced dietary plans, physical activity, and minimization of stress. Pediatric diabetes awareness campaigns and screening programs should be organized in community centers and schools for healthcare professionals to guide the parents of children with type 1 DM.

## Abbreviations

T1DM: Type 1 Diabetes mellitus

DKA: Diabetic Ketoacidosis

PICU: Pediatric Intensive Care Unit

HbA1c: Glycosylated Hemoglobin A1c

HR: Heart Rate

SBP: Systolic Blood Pressure

DBP: Diastolic Blood Pressure
